# Supplementary material for: Functional Characterization of the Dopaminergic Psychostimulant Sydnocarb as an Allosteric Modulator of the Human Dopamine Transporter
Source: Biomedicines. 2021 Jun 2;9(6):634. doi: 10.3390/biomedicines9060634 (PMC8227285; doi:10.3390/biomedicines9060634)
Supplement: Supplementary file 1 [file biomedicines-09-00634-s001.zip › biomedicines-1194997-supplementary.pdf]

<u>SUPPLEMENTARY INFORMATION</u>

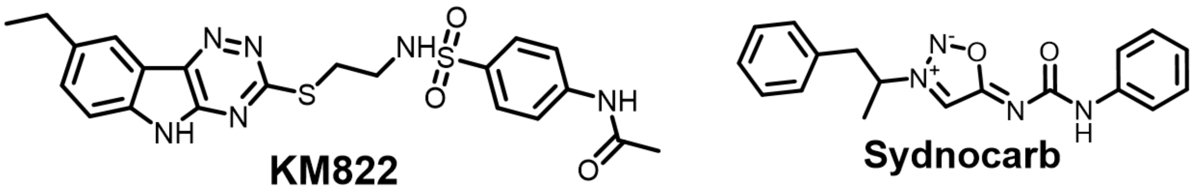

Figure S1. Chemical structures of KM822 and Sydnocarb.

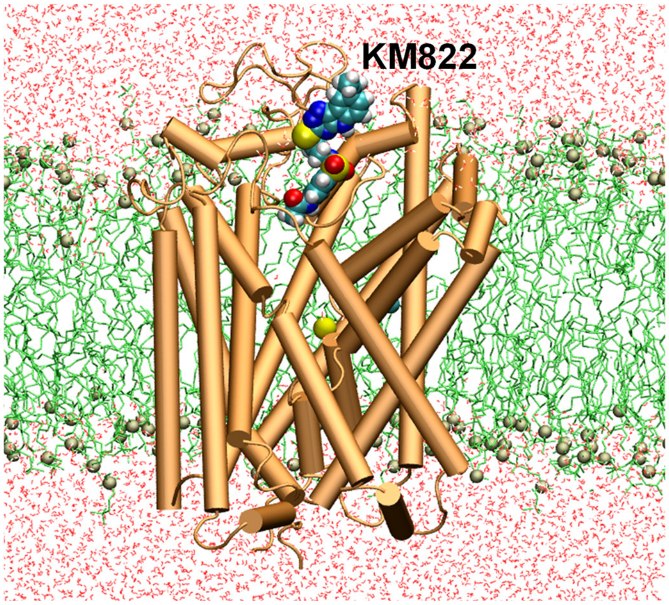

Figure S2. MD simulations of KM822 binding to human dopamine transporter in the outward-facing open conformer. The hDAT OFo conformer (orange) was embedded into membrane lipids (lime licorice) and solvated by 0.15M NaCl solution (not shown). A KM822 molecule (vDW format) is initially placed near the EC vestibule, at a similar site predicted in previous work [30].

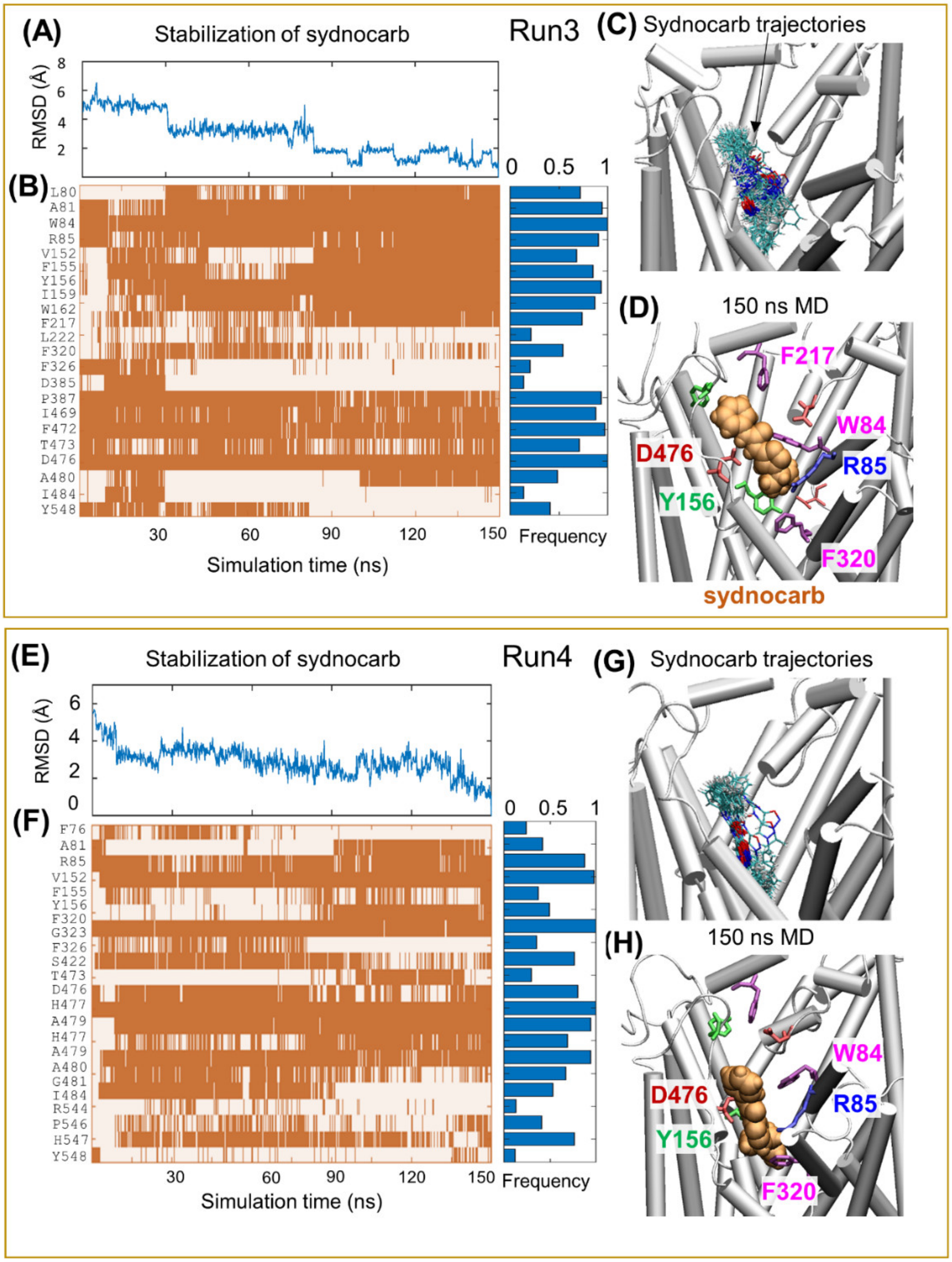

Figure S3: **MD simulations of sydnocarb in hDAT**. *Top* and *bottom* boxes display results from two independent runs Run3 and Run4. (**A)** and **(E)** Sydnocarb diffusion as a function time estimated by the RMSD of sydnocarb atoms with respect to final pose at 150 ns. (**B)** and **(F)** Time evolution of contacts (< 4.0Å closest atom-atom distance) between DAT and sydnocarb (indicated by *orange-shaded areas*) with binding frequency summarized by the horizontal *blue* bars on the *right panel.* (**C)** and **(G)** sydnocarb binding poses captured in simulations with a snapshot taken every 4 ns. The ligand conformations are shown in *cyan sticks*. (**D)** and **(H)** MD-resolved final poses of sydnocarb (*light orange* vDW), observed at the end of the MD simulation Run3 and Run4. Results for MD Run1 and Run2 can be found in **Figure 3**.

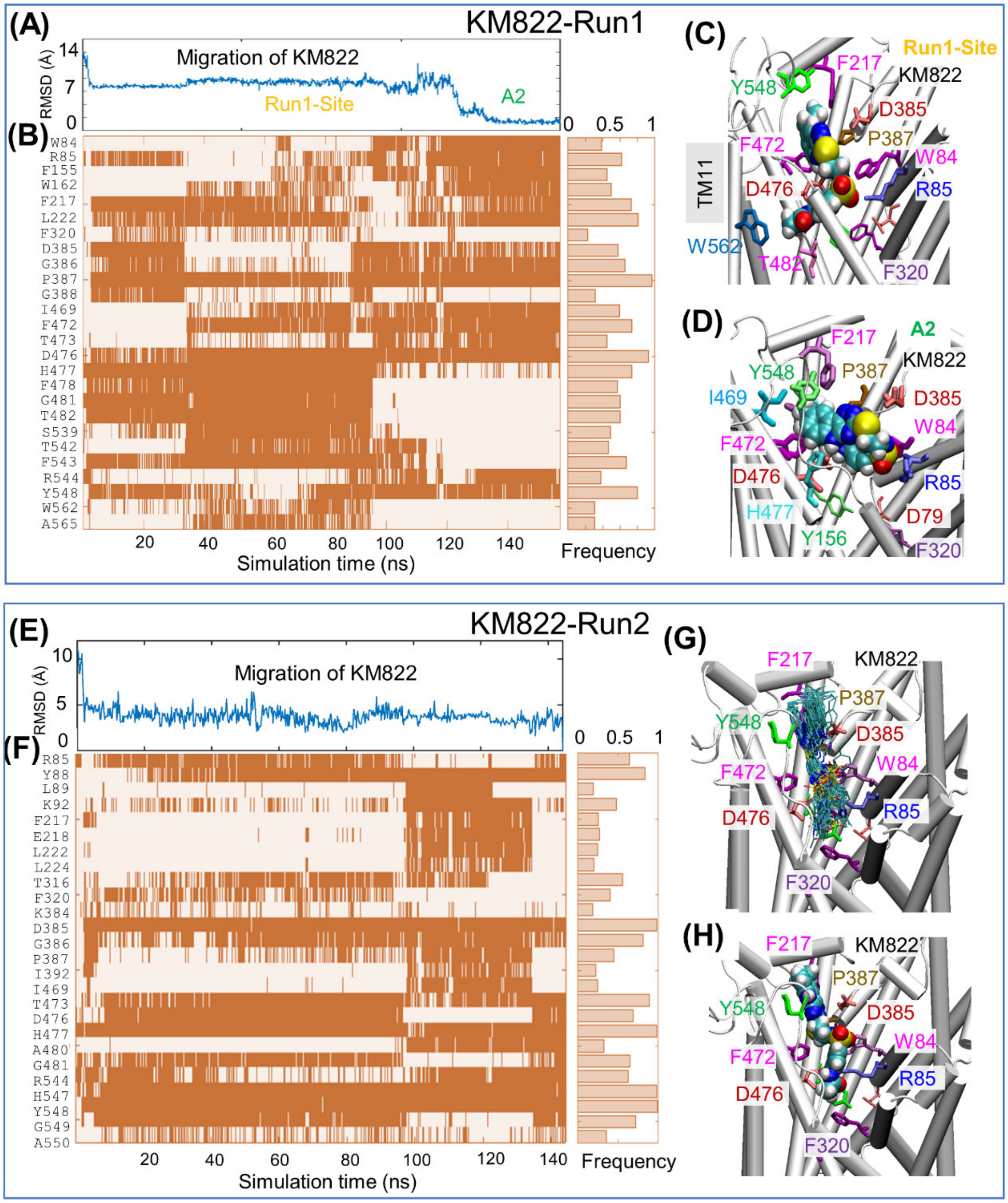

Figure S4: **MD simulations of KM822 binding to hDAT**. *Top* (Panels **A**-**D**) and *bottom* (Panels **E**-**H**) boxes display results from two independent runs KM822-Run1 and KM822-Run2. (**A**) and **(E)** KM822 diffusion as a function time estimated by the RMSD of KM822 atoms with respect to final pose at 150 ns. (**B)** and **(F)** Time evolution of contacts (< 4.0Å closest atom-atom distance) between DAT and KM822 (indicated by *orange-shaded areas*), with binding frequency summarized by the horizontal *light orange* bars on the *right panel.* (**C**) Representative Run1-Site binding pose of KM822 (vDW), captured transitionally in KM822-Run1. (**D**) and **(H)** MD-resolved final poses of KM822 (vDW), observed at the end of KM822-Run1 and KM822-Run2. *White, cyan, blue, red* and *yellow* spheres represent hydrogen, carbon, nitrogen, oxygen, and sulfur atoms, respectively, in KM822. (**G**) KM822 binding poses captured in the simulation KM822-Run2 with a snapshot taken every 4 ns. The ligand conformations are shown in *cyan sticks*.

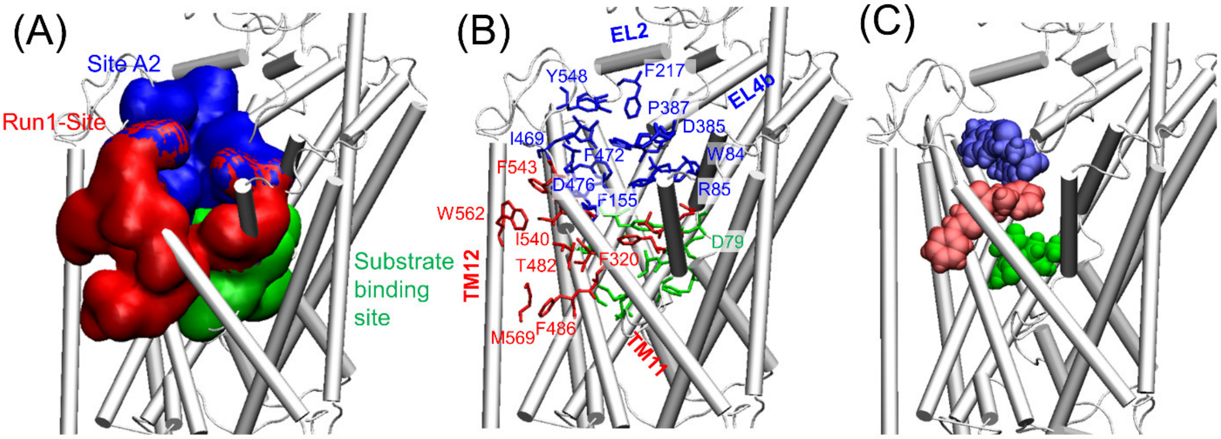

Figure S5: **Comparison of two allosteric sites observed for the binding of KM822 or sydnocarb with the substrate-binding site**. (**A**) Binding sites are shown in *green, red* and *blue* surface for the substrate-binding site, allosteric Site I and Site II, respectively. (**B**) Same binding sites are illustrated in *green, red* and *blue* sticks for residues composed of the substrate binding site, allosteric sites, A2 and Run1-Site, respectively. Run1-Site is displayed in *red* for residues commonly bound to KM822 and sydnocarb. A2 is shown in *blue* for residues commonly bound to KM822 and sydnocarb. Some residues such as W84/R85 and D476/H477 are shared between the two allosteric sites. Substrate-binding site composed residues in green are obtained from the previous simulations of cocaine binding to OF*o* DAT [38]. (**C**)Representative binding poses of ligand to the two sites shown in (**B**). *Green*, *red* and *blue* vDW balls represent cocaine, sydnocarb in the Run1_site, and KM822 in the A2 site.
